# Fault Identification Ability of a Robust Deeply Integrated GNSS/INS System Assisted by Convolutional Neural Networks

**DOI:** 10.3390/s19122734

**Published:** 2019-06-18

**Authors:** Xiaojun Zou, Baowang Lian, Peng Wu

**Affiliations:** 1 School of Electronics and Information, Northwestern Polytechnical University, Xi’an 710072, China; lian_bao_wang@163.com; 2 Department of integrated navigation, Xi’an Modern Control Technology Research Institute, Xi’an 710065, China; wupengrock@mail.nwpu.edu.cn

**Keywords:** deep GNSS/INS integration, fault identification, convolutional neural network, vector tracking loop

## Abstract

The problem of fault propagation which exists in the deeply integrated GNSS (Global Navigation Satellite System)/INS (Inertial Navigation System) system makes it difficult to identify faults. Once a fault occurs, system performance will be degraded due to the inability to identify and isolate the fault accurately. After analyzing the causes of fault propagation and the difficulty of fault identification, maintaining correct navigation solution is found to be the key to prevent fault propagation from occurring. In order to solve the problem, a novel robust algorithm based on convolutional neural network (CNN) is proposed. The optimal expansion factor of the robust algorithm is obtained adaptively by utilizing CNN, thus the adverse effect of fault on navigation solution can be reduced as much as possible. At last, the fault identification ability is verified by two types of experiments: artificial fault injection and outdoor occlusion. Experiment results show that the proposed robust algorithm which can successfully suppress the fault propagation is an effective solution. The accuracy of fault identification is increased by more than 20% compared with that before improvement, and the robustness of deep GNSS/INS integration is also improved.

## 1. Introduction

Modern navigation systems are developing in the direction of multi-sensor and deep information fusion. GNSS (Global Navigation Satellite System) has all-weather absolute positioning capability, but it is vulnerable to interference. On the contrary, INS (Inertial Navigation System), which has excellent anti-interference ability, can only provide relative position information. Thus, they have strong complementarity in performance. Deeply integrated GNSS/INS systems take into account the advantages of both GNSS and INS, and implement the deepest information fusion between GNSS and INS at present. Compared with loose integration and tight integration, deep GNSS/INS integration not only has better ability of anti-noise and anti-interference [1,2], but also can track weaker satellite signals [3] and acquire the temporarily occluded signals more quickly [4]. All of these advantages are attributed to the use of vector tracking loop [5]. The distinctive feature of vector tracking loop is that, after fusing GNSS and INS data, the navigation filter generates the locally predicted carrier frequency and code phase for each receiving channel to implement signal tracking. This kind of structure realizes the information exchange among the receiving channels, which is helpful for strong signals to assist the reception of weak signals. However, there is a serious problem that, when a receiving channel contains fault information, the information will spread to the remaining channels, thereby degrading the system performance. Therefore, timely detection and identification of faults are important for deep integration based on vector tracking loop.

Based on the approximate system model, a chi-square test is able to detect faults in real time by monitoring the difference between the measurements and the estimates of Kalman filter [6]. In order to weaken the influence of the inaccurate system model on the results of chi-square detection, Bando et al. propose a method of calculating the covariance of system model error in a stable period [7]. Rife et al. derive methods to generate rigorous integrity and continuity bounds for chi-square test when the signal is time-varying [8]. An integrated detection algorithm of combining particle filter and fuzzy inference system is presented by Bu et al. [9]. The residuals which are generated by particle filter are used as the inputs of fuzzy inference system to output the different anomaly levels. However, its performance is limited due to the high computational load of particle filter. Wavelet transformation which can extract characteristic values from fault data is a powerful mathematical tool for fault diagnostics owing to its multi-resolution capability [10]. Sun et al. study the method of detecting faults by employing the adaptive neural fuzzy inference algorithm [11], which allows real-time model-free residual analysis from Kalman filter estimates, and obtains better detection results than chi-square detection. The machine learning method SVM (Support Vector Machine) that has the ability to predict test statistics based on prior knowledge is applied in fault detection [12]. SVM cannot provide the accuracy of predicted value. However, another machine learning method named GPR (Gaussian Process Regression) is able to provide both the prediction of unknown input and the variance of prediction. The detection method of using GPR to predict the innovation of Kalman filter is shown to be especially effective in detecting gradual faults [13].

The above-mentioned methods can detect whether the GNSS receiver has faults or not, but they cannot identify the internal faults of GNSS. The whole GNSS is simply isolated from the integrated navigation system when an abnormality is detected in GNSS. In fact, the GNSS receiver contains multiple receiving channels to process satellite signals. In abnormal situations, the integrity of the integrated navigation system is maintained by just removing the fault channels from GNSS receiver, so that the system can keep working normally. 

The w-test method is often used to identify faults by checking whether the corresponding statistic follows the standard normal distribution or not [14]. RAIM (Receiver Autonomous Integrity Monitoring) which also makes the same binary hypothesis judgment as a w-test is extended to detect simultaneous multiple faults in GNSS/INS integrated systems [15]. The multiple solution separation (MSS) algorithm, which consists of a main estimator using all the available GPS (Global Positioning System) measurements and sub-estimators using different subsets of the GPS measurements, identifies faults by comparing the main solution with the different sub-solutions [16]. For deep integration, few researchers focus on the identification of GNSS fault channels. In [17], the pseudo-range and pseudo-range rate residuals outputted by each receiving channel are adopted as the monitoring objects to detect whether the channel is abnormal. In [18], a sub-filter based on Kalman filtering algorithm is proposed to be added to each receiving channel to identify faults. 

The above methods may be effective when the receiving channels are independent of each other. However, the situation is different in vector tracking loop. Due to the fault propagation problem, all receiving channels will be abnormal, resulting in the inaccurate identification of fault source. Thus, from the above analysis, it can be concluded that the inaccurate identification caused by fault propagation is lack of research, and there is no effective solution so far.

After analyzing the fault propagation path in deep integration, we find that maintaining correct navigation solution is the key to prevent fault propagation. The robust algorithm that can reduce the adverse effect of fault information on navigation solution is utilized in this study. It is derived by applying the maximum likelihood estimation to resist the outlier effect of measurement vector, and its function is realized by adjusting the covariance matrix of measurement noise with expansion factors [19]. The Huber weight function that is commonly used outputs the reciprocal of normalized residual as an expansion factor when the residual value is larger than the preset threshold [20,21]. However, its performance is restricted by fixed parameter value and limited parameter range. The IGGIII (Institute of Geodesy and Geophysics III) scheme is another frequently used method to generate expansion factors [22,23], but it has more parameters to configure, and has the same problem as Huber. In [24], the fuzzy inference algorithm is proposed to determine the value of expansion factor. By comparing the difference between the theoretical covariance and the actual covariance of residuals, the expansion factor is adjusted adaptively. Although fuzzy inference rules can be acquired from prior knowledge or experience, the exact membership function is difficult to be defined. Thus, the output of the fuzzy inference algorithm is not guaranteed to be the optimal value. 

The neural network is famous for its strong self-learning ability and complex system mapping ability, and it has the potential to generate expansion factors. CNN (convolutional neural network) is a kind of deep neural network, and has achieved great success in the field of image processing [25,26]. Moreover, because of the attractive qualities, it has been gradually applied in the field of speech recognition [27], behavior recognition [28], and fault mode recognition [29,30]. Compared with the traditional multilayer perceptual neural network (MLP), CNN can deal with more complex classification problems for its deeper network structure. In the neural network, the number of neurons is greatly reduced by the pooling operation, and the number of parameters which are needed to be trained is also reduced by sharing weights. Thus, CNN has the better generalization ability and the lower network complexity [31]. After sufficient training, CNN is able to output the desired value that meets the error precision requirement, and adaptively adjust the output according to the changes in the environment.

In this paper, a novel robust algorithm based on CNN is proposed to solve the fault propagation problem. By utilizing CNN, the optimal expansion factor of the robust algorithm is obtained adaptively according to the changes of fault. In addition, the performance of the proposed robust algorithm that is not restricted by parameter range is superior to the traditional robust algorithm. As a result, the adverse effect of fault data on navigation solution is weakened, and the propagation of fault is prevented. Eventually, the accuracy of fault identification is improved after solving the fault propagation problem.

The rest of this paper is organized as follows: Section 2 analyses the root cause of fault propagation and the difficulty of fault identification. In Section 3, it introduces the method of using the robust algorithm to prevent the propagation of fault information, and describes the robust algorithm based on CNN. Experiment setup and result analysis are provided in Section 4. In addition, the content of this paper is summarized in Section 5.

## 2. Fault Identification Method for Deep Integration

### 2.1. The Cause of Fault Propagation

**Figure 1:**
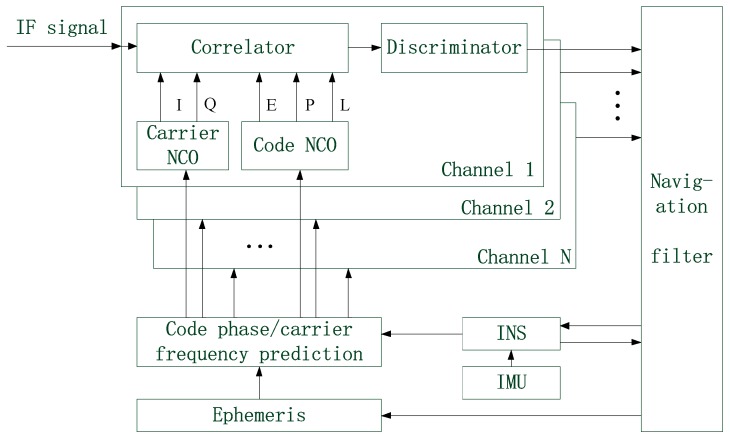
The structure of deep GNSS (Global Navigation Satellite System)/INS (Inertial Navigation System) integration.

In the deeply coupled GNSS/INS integrated system, the vector tracking loop is a feedback loop. GNSS IF (Intermediate Frequency) data are processed by multiple receiving channels and then put into the navigation filter. After fusing all the received data in a centralized manner, the navigation filter outputs the positioning errors to correct INS. Next, the carrier frequency and code phase of each receiving channel that are predicted by making use of both the INS data and ephemeris are used to configure the corresponding NCO (Numerically Controlled Oscillator). The structure of deep GNSS/INS integration is shown in Figure 1.

The navigation filter is based on the Extended Kalman Filtering algorithm, and the system state is defined as

(1) $$\Delta X={\left[\Delta R^{e},\Delta V^{e},\Delta \varphi^{e},\Delta W^{b},\Delta A^{b},\Delta C\right]}^{T},$$

where ΔR represents position error vector, ΔV represents velocity error vector, Δφ represents attitude error vector, ΔW represents the bias error of gyroscope, and ΔA represents the bias error of accelerometer. $\Delta C=\left[\Delta t_{b},\Delta t_{d}\right],$ $\Delta t_{b}$ and $\Delta t_{d}$ represent the clock bias error and clock drift error of GNSS receiver, respectively. The superscripts e and b represent the ECEF (Earth-Centered Earth-Fixed) coordinate system and the body coordinate system, respectively.

Pseudo-range error and pseudo-range rate error are adopted as the measurements of navigation filter, and then the measurement vector at time k is expressed as

(2) $$Z_{k}^{\mathrm{Nav}}={\left[\Delta \rho_{1},\Delta {\dot{\rho }}_{1},\Delta \rho_{2},\Delta {\dot{\rho }}_{2},\ldots \ldots ,\Delta \rho_{N},\Delta {\dot{\rho }}_{N}\right]}^{T},$$

where $\Delta \rho_{i}$ and $\Delta {\dot{\rho }}_{i}$ represent pseudo-range error and pseudo-range rate error of the ith receiving channel, respectively. N is the number of receiving channels.

$\Delta \rho_{i}$ is obtained from code phase error $\Delta \tau_{i}$, and $\Delta {\dot{\rho }}_{i}$ is obtained from carrier frequency error $\Delta f_{i}$. Their expressions are shown in Equations (3) and (4), respectively:

(3) $$\Delta \rho_{i}=\frac{c}{f_{i,code}}\Delta \tau_{i},$$

(4) $$\Delta {\dot{\rho }}_{i}=\frac{c}{f_{i,carr}}\Delta f_{i},$$

where $f_{i,code}$ denotes code frequency, $f_{i,carr}$ denotes carrier frequency, and c denotes the speed of light.

Extended Kalman Filter (EKF) adopts an iterative method to estimate the system state, including two processes of prediction and correction.

Prediction:

(5) $$\Delta X_{k|k-1}=F\Delta X_{k-1},$$

(6) $$P_{k|k-1}=FP_{k-1}F+Q,$$

Correction:

(7) $$W_{k}=P_{k|k-1}H_{k}^{T}{\left(H_{k}P_{k|k-1}H_{k}^{T}+R\right)}^{-1},$$

(8) $$\Delta X_{k}=\Delta X_{k|k-1}+W_{k}\left(Z_{k}^{\mathrm{Nav}}-H_{k}\Delta X_{k|k-1}\right),$$

(9) $$P_{k}=\left(I-W_{k}H_{k}\right)P_{k|k-1},$$

where $P_{k}$ is the covariance matrix of system state error, $W_{k}$ is the gain matrix, Q is the covariance matrix of process noise, and R is the covariance matrix of measurement noise. $H_{k}$ is the measurement matrix whose coefficients are unit vectors in the line-of-sight direction from the receiver antenna to satellites. F is the state transition matrix, whose coefficients are available in [32].

After corrected by the system state of navigation filter, INS output the navigation solution. Combining navigation solution and ephemeris data, carrier frequency, code frequency and code phase of the ith satellite signal can be predicted. In [33], their expressions are defined as follows:

(10) $${\hat{f}}_{i,\mathrm{carr}}=f_{\mathrm{IF}}-\frac{f_{\mathrm{sa}}}{c}\left[\left({\dot{x}}_{i}-\dot{x}\right)\alpha_{x,i}+\left({\dot{y}}_{i}-\dot{y}\right)\alpha_{y,i}+\left({\dot{z}}_{i}-\dot{z}\right)\alpha_{z,i}-t_{d}\right],$$

(11) $${\hat{f}}_{i,\mathrm{code}}=f_{\mathrm{code}}-\frac{f_{\mathrm{code}}}{c}\left[\left({\dot{x}}_{i}-\dot{x}\right)\alpha_{x,i}+\left({\dot{y}}_{i}-\dot{y}\right)\alpha_{y,i}+\left({\dot{z}}_{i}-\dot{z}\right)\alpha_{z,i}-t_{d}\right],$$

(12) $${\hat{\varphi }}_{i,k+1}={\hat{\varphi }}_{i,k}-\frac{1}{c}\left[\left(\delta x_{i}-\delta x\right)\alpha_{x,i}+\left(\delta y_{i}-\delta y\right)\alpha_{y,i}+\left(\delta z_{i}-\delta z\right)\alpha_{z,i}-\delta t_{b}\right],$$

where $f_{\mathrm{IF}}$ is the intermediate frequency, $f_{\mathrm{sa}}$ is the frequency of satellite signal, $f_{\mathrm{code}}$ is the frequency of pseudo code, $\left[{\dot{x}}_{i},\;{\dot{y}}_{i},\;{\dot{z}}_{i}\right]$ is the velocity vector of the ith satellite, and $\left[\dot{x},\;\dot{y},\;\dot{z}\right]$ is the velocity vector of receiver. $\left[\delta x_{i},\delta y_{i},\delta z_{i}\right]$ and [δx,δy,δz] are the position variations of the ith satellite and the receiver from time k to k+1, respectively. $\Delta t_{b}$ is the clock bias variation of receiver from time k to k+1, and $t_{d}$ is the clock drift error of receiver. $\left[\alpha_{x,i},\alpha_{y,i},\alpha_{z,i}\right]$ is the unit vector in the line-of-sight direction from receiver antenna to the ith satellite.

When an abnormality occurs in $Z_{k}^{\mathrm{Nav}}$, the value of ΔX is contaminated according to Equation (8). After corrected by ΔX, INS output a wrong navigation solution. According to Equations (10)–(12), the navigation solution is shared by all visible satellites. Then, the wrong navigation solution leads to deviations in the predicted receiving parameters of all satellite signals. Thus, no matter which satellite is abnormal, its errors will spread to other receiving channels, spoiling the reception of satellite signals. Moreover, the errors will spread to each other continually. The navigation system eventually loses lock on all satellite signals with the accumulation of errors.

### 2.2. Fault Identification Method

If only the pseudo-range errors in $Z_{k}^{\mathrm{Nav}}$ are considered, i.e., $Z_{k}={\left[\Delta \rho_{1},\Delta \rho_{2},\ldots \ldots ,\Delta \rho_{N}\right]}^{T}$, the expressions of innovation $V_{k}$ and its covariance matrix $A_{k}$ defined in [34] are

(13) $$V_{k}=\;Z_{k}-H_{k}\Delta X_{k|k-1},$$

(14) $$A_{k}=E\left[V_{k}V_{k}^{T}\right]=H_{k}P_{k|k-1}H_{k}^{T}+R,$$

where the dimension of $H_{k}$ needs to be modified to N×17.

The innovation $V_{k}$ is assumed to be Gaussian white noise with a zero mean in the fault free case [35]. However, if a fault occurs, a large deviation will arise between the measurements and the estimates of $Z_{k}$. At this moment, the mean value of $V_{k}$ is greater than zero, so it can be used as the rule for fault detection.

According to the w-test method, the fault identification statistic is constructed as:

(15) $$w_{i,k}=\left|\frac{-e_{i}^{T}V_{k}}{\sqrt{e_{i}^{T}A_{k}e_{i}}}\right|,$$

where $e_{i}={\left[0,\ldots \ldots ,0,1,0,\ldots \ldots ,0\right]}^{T}$. It represents that the value of the ith component is 1, and the rest are 0.

When there is no fault, $w_{i,k}$ should follow the standard normal distribution, i.e., $w_{i,k}\sim N\left(0,1\right)$.

Given the false alarm probability $P_{\mathrm{fa}}$, if

(16) $$w_{i,k}>N_{1-P_{\mathrm{fa}}/2}\left(0,1\right),$$

then the ith channel is considered to be faulty; otherwise, it is normal.

From the above analysis, the statistic $w_{i,k}$ is related to $\Delta \rho_{i}$. If there is an abnormality in $\Delta \rho_{i}$, $w_{i,k}$ will exceed the alarm threshold. As described in Section 2.1, the fault information is propagated mutually among the receiving channels in the vector tracking loop. Errors in one channel can spread to all other channels, resulting in abnormal measurements in all channels. Thus, the actual fault channel will not be distinguished for the reason that the identification statistics of all channels exceed the alarm threshold.

## 3. Robust Algorithm Based on CNN

### 3.1. The Principle of Robust Algorithm

The problem of fault propagation not only makes all receiving channels work abnormally, but also increases the difficulty of fault identification, making the deep GNSS/INS integration extremely vulnerable to fault. In real life, many types of faults are likely to be encountered, such as jamming, device aging, and occlusion. Therefore, it is necessary to solve the fault propagation problem to improve fault diagnosis capability.

The basis of fault propagation is that the parameters of all receiving channels are predicted based on the same navigation solution. Once the navigation solution is wrong, the reception of all channels will be abnormal. Thus, the key of solving the problem is to maintain the correctness of navigation solution. The robust algorithm, which can reduce the adverse effect of fault data on navigation solution by adjusting the parameters of navigation filter, is a promising solution.

In EKF, $\Delta X_{k|k-1}$ and $\Delta X_{k}$ are the prediction and estimation of $\Delta X_{k-1}$, respectively. Then, the prediction error of state vector is expressed as

(17) $$E_{k}=\Delta X_{k}-\Delta X_{k|k-1}.$$

The expression of residual $L_{k}$ is

(18) $$L_{k}=Z_{k}^{\mathrm{Nav}}-H_{k}\Delta X_{k}.$$

If the measurement $Z_{k}^{\mathrm{Nav}}$ contains gross errors, the minimum condition is constructed as

(19) $$\sigma =L_{k}^{T}{\tilde{R}}^{-1}L_{k}+E_{k}^{T}P_{k|k-1}^{-1}E_{k}=min,$$

where $\tilde{R}$ is the covariance matrix of $L_{k}$, and $P_{k|k-1}$ is the covariance matrix of $E_{k}$.

In [36], the solution of Equation (19) is

(20) $$\Delta {\tilde{X}}_{k}=\Delta X_{k|k-1}+P_{k|k-1}H_{k}^{T}{\left(H_{k}P_{k|k-1}H_{k}^{T}+\tilde{R}\right)}^{-1}\left(Z_{k}^{\mathrm{Nav}}-H_{k}\Delta X_{k|k-1}\right).$$

Comparing $\Delta {\tilde{X}}_{k}$ with $\Delta X_{k}$ in Equation (8), the difference is that the matrix R is replaced by $\tilde{R}$. It shows that, when there are gross errors in the measurement, the corresponding solution is obtained by adjusting R. Matrix R, as the covariance matrix of measurement error, reflects the severity of errors. If the measurements are unreliable, the proportion of measurements in the filtering should be reduced, and the value of R should be enlarged. However, it should be emphasized that, in order to avoid changing the results of fault identification, the matrix R in Equation (14) does not need to be adjusted. 

If the members of $Z_{k}^{\mathrm{Nav}}$ are independent of each other, $\tilde{R}$ can be regarded as a diagonal matrix, i.e., $\tilde{R}=diag\left\{{\tilde{R}}_{1},{\tilde{\dot{R}}}_{1},{\tilde{R}}_{2},{\tilde{\dot{R}}}_{2},\ldots \ldots ,{\tilde{R}}_{N},{\tilde{\dot{R}}}_{N}\right\}$. As shown in Equation (21), ${\tilde{R}}_{i}$ is obtained by multiplying $R_{i}$ and the expansion factor $\alpha_{i}$ together:

(21) $${\tilde{R}}_{i}=\alpha_{i}R_{i},$$

where $\alpha_{i}\geq 1$. ${\tilde{\dot{R}}}_{i}$ is calculated in the same way as ${\tilde{R}}_{i}$.

Expansion factor is generated by CNN in this study, and the generation method will be described in the following section. 

### 3.2. The Structure of CNN

**Figure 2:**
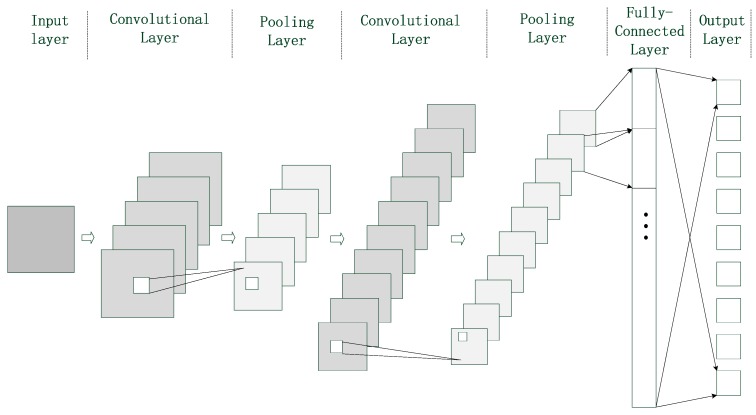
The typical structure of convolutional neural network (CNN).

CNN which is inspired by the structure of nerve cells in the visual cortex extracts and classifies image information through a multilayer structure. As shown in Figure 2, the structure composed of seven layers is the basic form of CNN. In order to achieve better performance, CNN usually contains more layers. A pair of convolutional layer and pooling layer is a group. According to the needs of practical application, many groups could be added.

In Figure 2, the input layer receives the sample data and preprocesses the data such as normalization. Then, it packages the data according to the input format to prepare for the next operation. The output layer provides the probabilities corresponding to different results. Convolutional layer, pooling layer and fully-connected layer are the decisive parts of CNN. Their structures are much more complex than that of the two above-mentioned layers, so they are described as follows one by one.

The convolutional layer extracts different levels of image features by convolutional operation. The higher the level of convolutional layer is, the finer the image features are extracted. It is composed of several feature planes, each of which shares a convolution kernel with one of the feature planes of the upper layer. If the upper layer of the current convolutional layer contains multiple feature planes, there will be multiple convolution kernels. The feature plane of convolutional layer is equal to the sum of convolution between multiple feature planes and convolutional kernels. Convolutional kernel is a weight matrix whose coefficients are adjusted during the training period of CNN. The input–output relationship of convolutional layer is expressed as:

(22) $$Y_{j}=f_{\mathrm{cov}}\left(\sum_{i=1}^{M}\mathrm{cov}\left(X_{i},W_{i,j}\right)+b_{j}\right),$$

where $Y_{j}$ represents the jth feature plane of convolutional layer. $X_{i}$ represents the ith feature plane, which belongs to the upper layer of the current convolutional layer. $W_{i,j}$ represents the coefficients of convolutional kernel between $X_{i}$ and $Y_{j}$. $b_{j}$ is the bias. M is the number of $X_{i}$. The function cov(⋅) represents the convolutional operation, and $f_{\mathrm{cov}}\left(\cdot \right)$ represents the nonlinear activation function of convolutional layer.

The function of pooling layer is to reduce the number of connections between two convolutional layers. Through the subsampling operation, some redundant information in the image is removed, and the main feature of image is obtained. The feature planes of pooling layer and convolutional layer correspond one to one, and the number of planes is the same. Average pooling and max pooling are the commonly used methods of subsampling. Average pooling takes the average of sampling area as the output, while max pooling chooses the maximum of sampling area as the output. The output of pooling layer is as follows:

(23) $$S_{j}=f_{\mathrm{pool}}\left(Y_{j}\right),$$

where $S_{j}$ represents the output of the jth feature plane of pooling layer, and the function $f_{\mathrm{pool}}\left(\cdot \right)$ represents the subsampling operation.

Fully-connected layer lies between pooling layer and output layer. It integrates the local features which are extracted from pooling layer, and transfers the integrated results to the output layer. The relationship between input and output in fully-connected layer is expressed by Equation (24):

(24) $$l_{j}=f_{\mathrm{fc}}\left(\sum_{i=1}^{L}v_{i,j}c_{i}+\varepsilon_{j}\right),$$

where $l_{j}$ represents the jth output of fully-connected layer. $c_{i}$ represents the ith neuron of fully-connected layer. $v_{i,j}$ denotes the connection coefficient between $c_{i}$ and $l_{j}$. $\varepsilon_{j}$ denotes the bias. $f_{\mathrm{fc}}\left(\cdot \right)$ represents the nonlinear activation function of the fully-connected layer. 

### 3.3. Configuration of CNN

In this study, the mean values and standard deviations of residuals are the inputs of CNN, and the probabilities of the corresponding expansion factors are the outputs. The input data are normalized at first, and then divided into data segments with size of 2 × 10. Each data segment that acts like a picture is the processing object of CNN. Two rows of elements in the data segment correspond to the mean values and standard deviations of residuals, respectively. The data length is set to 10 to allow for both short data processing time and sufficient data quantity.

**Figure 3:**
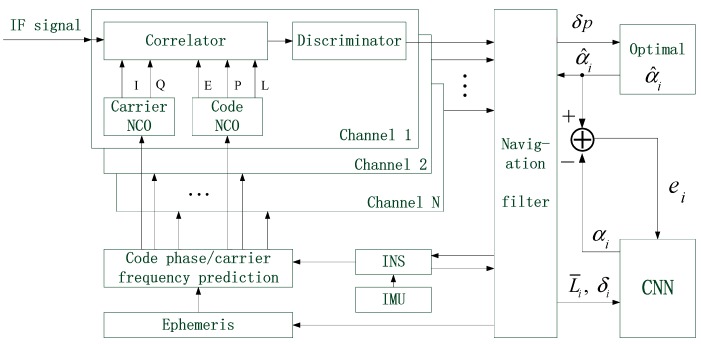
The system structure during the training stage of CNN.

The residual of each receiving channel contains two components: pseudo-range residual and pseudo-range rate residual, which correspond to different coefficients of $\tilde{R}$. Thus, two CNNs are required for each receiving channel. One CNN is for pseudo-range residuals, and the other is for pseudo-range rate residuals. CNN needs to be trained before being put into use. During the training stage of CNN, the system structure is shown as follows:

In Figure 3, the pseudo-range residual of the ith receiving channel is shown as an example. ${\bar{L}}_{i}$ and $\delta_{i}$ denote the mean and standard deviation of residuals, respectively. Abnormal values are artificially added in the pseudo-range error of the ith receiving channel, and then the value of $R_{i}$ is adjusted by different expansion factor. When the positioning error δp is minimized, the optimal expansion factor ${\hat{\alpha }}_{i}$ is obtained. Next, CNN starts to be trained. ${\hat{\alpha }}_{i}$ is compared with the expansion factor $\alpha_{i}$, which is predicted by CNN. The smaller the difference between the two factors, the more accurate the prediction of CNN is. The training goal of CNN is to minimize the difference. Actually, the outputs of CNN are expressed in probability. Different expansion factors correspond to different probabilistic values. At last, the alternative value corresponding to the maximum probability is determined as the expansion factor of CNN. The loss function is constructed as:

(25) $$e\left(W,b\right)=\frac{1}{N_{e}}\sum_{j=1}^{N_{e}}{\left(o_{j}-{\hat{o}}_{j}\right)}^{2},$$

where $o_{j}$ represents the jth output of CNN, and ${\hat{o}}_{j}$ represents the expected output. Both $o_{j}$ and ${\hat{o}}_{j}$ are probabilistic values ranging from 0 to 1. $N_{e}$ represents the number of CNN output. The expected probability corresponding to ${\hat{\alpha }}_{i}$ should be equal to 1, and the others should be equal to 0. 

In the training process, back-propagation is applied to update the trainable parameters in the neural network by minimizing the loss function [37]. The parameters include the weights of convolution kernel and the connection coefficients of fully connected layer. Back-propagation is implemented based on the stochastic gradient algorithm, so the method of updating the parameters is as follows:

(26) $$W_{k+1}=W_{k}-\eta \frac{\partial e\left(W_{k},b_{k}\right)}{\partial W_{k}},$$

(27) $$b_{k+1}=b_{k}-\eta \frac{\partial e\left(W_{k},b_{k}\right)}{\partial b_{k}},$$

where η denotes the learning rate. $W_{0}$ and $b_{0}$ are initialed with small random values.

In order to prevent over-fitting in the training process, some measures are taken to increase the sparsity and randomness of neural network. Firstly, the input and output data of CNN are normalized. In addition, a weight decay is adopted to limit the growth of the weights and suppress the effects of static noise [38]. It adds a term in the updating procedure of weights, as shown in Equations (28) and (29):

(28) $$W_{k+1}=W_{k}-\eta \left(\frac{\partial e\left(W_{k},b_{k}\right)}{\partial W_{k}}+\beta W_{k}\right),$$

(29) $$b_{k+1}=b_{k}-\eta \left(\frac{\partial e\left(W_{k},b_{k}\right)}{\partial b_{k}}+\beta b_{k}\right).$$

In addition, dropout is applied in full-connected layer. It drops neurons and their connections from the neural network randomly. For the input data, the network is different owing to the randomness of dropout. This prevents neurons from co-adapting too much and obtains a better generalization ability [39]. 

In order to output the corresponding expansion factor adaptively according to the fault value, CNN must be trained by a large number of fault data. The training data of CNN are composed of the mean values and standard deviations of residuals. The range of residual value is related to the severity of fault, and the standard deviation of residual is related to the carrier to noise density ratio (C/No) of signal. When fault values vary from 0 to 100 m, the range of pseudo-range residuals is the same as that of fault values. Assuming that the C/No varies from 30 to 50 dB-Hz, the standard deviation ranges from 0 to 12. The range of pseudo-range residuals is divided into ten regions, and 10,000 random numbers are generated in each region denoted by $p_{i}$ (where i ranges from 0 to 9). Meanwhile, the range of standard deviation is divided into four regions, and 10,000 random numbers are also generated in each region denoted by $q_{j}$ (where j ranges from 0 to 3). By combining $p_{i}$ and $q_{j}$, forty sets of data which correspond to forty different combinations are obtained. Each dataset includes 1000 arrays of two rows and ten columns. Before being used, these forty datasets are normalized. In addition, they are labeled by forty tags, which are used as the expected output of CNN. 

In practical application, due to the interference of noise and abnormalities, there will be gross errors in residuals. When processing such data, CNN outputs the probabilities that the data belong to each combination, and finally chooses the combination corresponding to the maximum probability as the result of data recognition. It indicates that this recognition method which can filter out gross errors is robust. 

CNN is designed to have seven layers, including two convolutional layers and two pooling layers. Average pooling is applied in two pooling layers. Sigmoid function is used as the activation function for both convolutional layer and full-connected layer. Since the size of CNN input data is 2 × 10, the size of convolutional kernel and the subsampling size of pooling layer are specially designed to be compatible with this type of data. The detailed configuration information of CNN structure is shown in Table 1. In addition, 3@2 × 5 denotes that the convolutional layer C1 contains three convolutional kernels with size of 2 × 5, while 3@2 × 6 denotes three feature planes with size of 2 × 6, which are results of convolution between input layer and convolutional kernels. In the pooling layers P1 and P2, only the column elements are sampled with the step length of 2. NA is the abbreviation for “not applicable”. The batch size is 50, and the number of epochs is 100.

**Figure 4:**
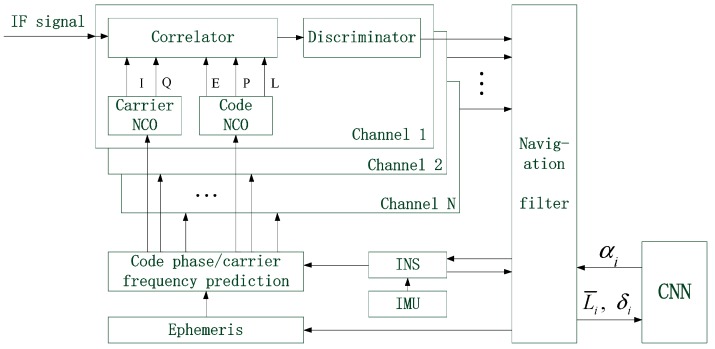
The structure of deep integration assisted by CNN.

After completing the training, CNN needs to be tested to check whether its output is correct. If the error ratio is less than the required error accuracy 0.05, it indicates that the training requirement is met; otherwise, further training is needed. Once the training goal is reached, the structure and coefficients of CNN are fixed, and then it can be put into use. The structure of deep integration assisted by CNN is shown in Figure 4. 

**Figure 5:**
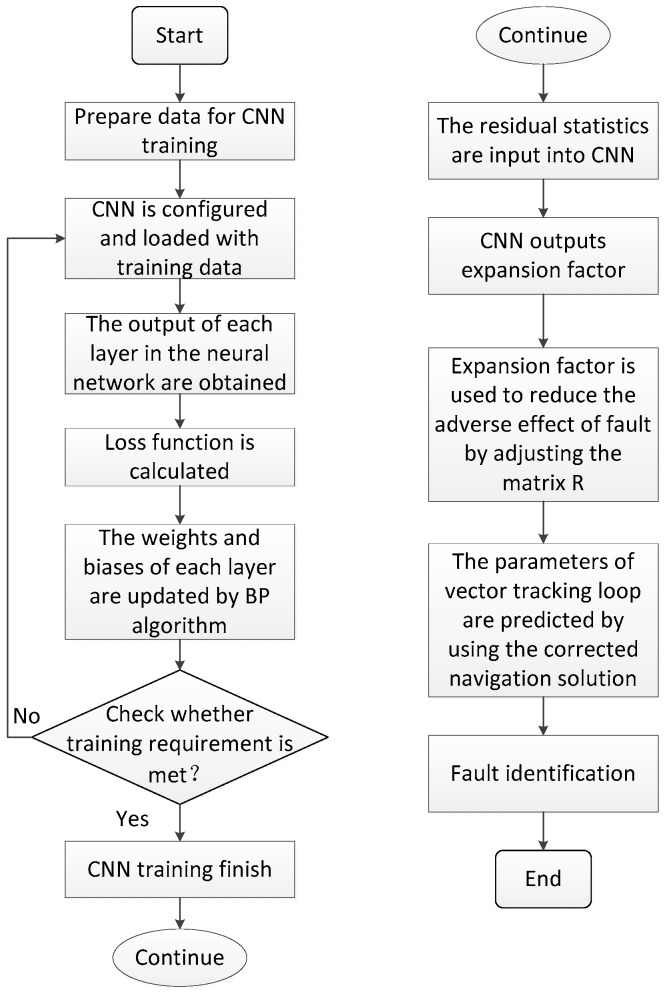
Flowchart of the proposed method.

Figure 5 shows the complete flowchart of the proposed method. It mainly consists of two parts: one part is the training process of CNN, and the other is the method of fault identification using the robust algorithm based on CNN.

## 4. Experiment Setup and Result Analysis

### 4.1. Experiment Setup

**Figure 6:**
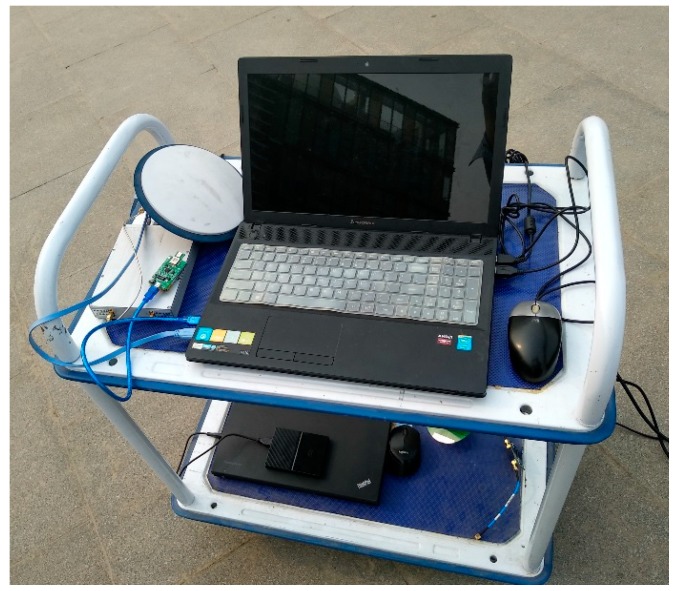
Experimental equipment.

GNSS IF data and MEMS (Micro-Electro-Mechanical System) data are simultaneously input into the Matlab based software receiver to complete GNSS signal tracking and the navigation solution calculation. Taking into account both the number of satellites and computational complexity, the software receiver is designed to have eight receiving channels. Figure 6 shows the composition of experimental system. The parameters of MEMS are shown in Table 2. GPS L1 signals are sampled as GNSS data with the IF signal sampler SPL-SIS800, of which the IF is 4.02 MHz and the sampling frequency is 20 MHz. The integration time of correlator is set to 10 ms, and the output rate of navigation solution result is 10 Hz. To identify faults, the false alarm probability is set to 0.02, and the corresponding alarm threshold is 2.58.

In practical application, there are many reasons for the fault of GNSS and INS. Some types of fault data are easy to be obtained, such as occlusion leading to temporary loss of GNSS signals, etc. However, it is difficult for most fault types. Thus, the commonly used method is to artificially inject abnormal values into the collected data to simulate faults. In this study, the experiment includes two parts: one is simulation test, and the other is experimental test. The simulation test is to simulate different fault modes by injecting various types of abnormal values, and obtain the accuracy of fault identification by constructing a large number of fault data, while the experimental test adopts the real fault data which are collected from outdoors during multiple satellites are occluded.

### 4.2. Result Analysis

#### 4.2.1. Simulation Test

Considering that the possibility of simultaneous multiple satellite faults in real life is very small, so only one satellite is injected with fault in the simulation test, and the fault will persist until the end of simulation. The injected faults are classified into two types: one is the gradual fault with the abnormal values increasing gradually, and the other is the abrupt fault with a step error appearing at a certain time. The measurements are the only input of navigation filter, thus the appropriate object of fault injection is pseudo-range error, which is a component of the measurements. GPS data are collected outdoors in a static situation.

A ramp error of slope 0.2 m/s is added in the pseudo-range error of satellite 14 to simulate the gradual fault. The start time of fault injection is 150 s.

**Figure 7:**
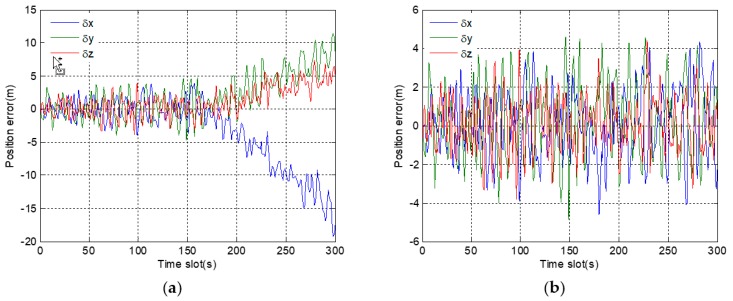
Position errors in 3D direction corresponding to gradual fault: (**a**) before using a robust algorithm; (**b**) after using a robust algorithm.

In Figure 7a, position errors in a three-dimensional direction are within the normal range before fault injection. However, after 150 s, position errors gradually deviate, and the deviations from the correct position are getting larger and larger. However, in Figure 7b, even after a long time, there are not obvious deviations in the position errors after fault injection. The results show that, under the adjustment of the expansion factor, the adverse effect of fault information on the positioning results is basically eliminated.

The variances of position error $\delta p\;\left(\delta p=\sqrt{\delta x^{2}+\delta y^{2}+\delta z^{2}}\right)$ corresponding to different methods are shown in Table 3. The function Huber only has a configurable parameter d, which is a constant ranging from 1.0 to 1.5. For the robust algorithm based on Huber, different variances are obtained by changing the value of parameter d, indicating that the parameter value should be set carefully. Moreover, the parameter value is limited to a small range, which also restricts the performance of Huber. However, the robust algorithm based on CNN is more flexible without the limitation of parameter range. In addition, the proposed robust algorithm is proved to be effective by the result that its variance is close to that without fault. However, CNN has a complex structure and many convolutional operations, so its computation is time-consuming. Both the robust algorithm based on CNN and Huber are tested on a desktop computer equipped with the CPU Intel i5-2400. When processing the same dataset with size of 2 × 40,000, CNN spends 36.21 s, but Huber spends 0.85 s. Moreover, due to processing one data block at a time, CNN may be unable to respond to data changes in time. The larger the data block, the more data processing time it takes. Thus, CNN is not suitable for the applications with high real-time requirement.

**Figure 8:**
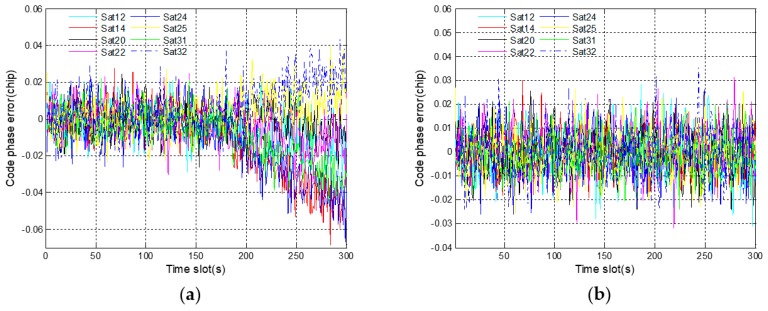
Code phase errors of eight satellite signals corresponding to gradual fault: (**a**) before using a robust algorithm; (**b**) after using a robust algorithm.

Figure 8a shows the code phase errors of eight receiving channels. One satellite corresponds to one receiving channel, and satellite 14 corresponds to channel 2. Starting from 150 s, different deviations gradually occur in the code phase errors of all eight satellites. This phenomenon shows that the fault information of channel 2 has spread to the other receiving channels. Considering the results in Figure 7a, it is the incorrect positioning results that lead to inaccurate code phases. In Figure 8b, the code phase errors of eight satellites always stay normal even after fault injection. The propagation of fault information is successfully suppressed by the robust algorithm. In addition, since the fault injection object is a pseudo-range error rather than a code phase error, the code phase of satellite 14 is not contaminated by fault. On the contrary, this code phase is accurate owing to the prediction based on corrected navigation solution.

**Figure 9:**
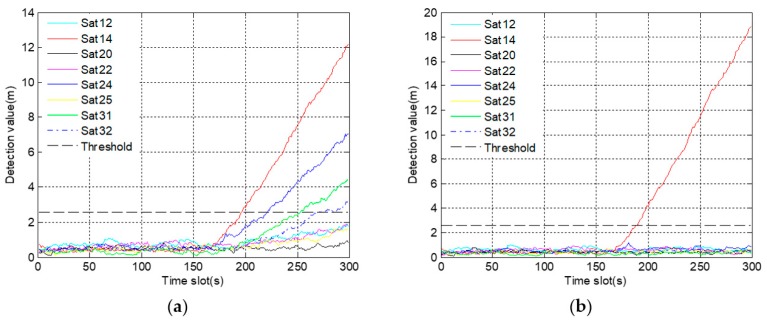
The graph of fault identification statistics corresponding to gradual fault: (**a**) before using a robust algorithm; (**b**) after using a robust algorithm.

In Figure 9a, there are four satellites, whose detection results exceed the alarm threshold due to the fault propagation problem. The results make it difficult not only to determine the number of fault satellites, but also to distinguish the actual fault satellite. In Figure 9b, only the detection values of satellite 14 gradually exceed the alarm threshold after 150 s. Thus, it is easy to confirm that satellite 14 is the only fault satellite. For further comparison, the Monte Carlo test is carried out. The accuracy of fault identification is increased from 77% to 98% after improvement. It indicates that employing a robust algorithm is beneficial to identify the gradual fault.

The second test is to inject the abrupt fault into the collected data. In order to facilitate the comparison with a gradual fault, satellite 14 is still the object of fault injection. The injection method is to add an outlier of 30 m in the pseudo-range error from 150 s.

**Figure 10:**
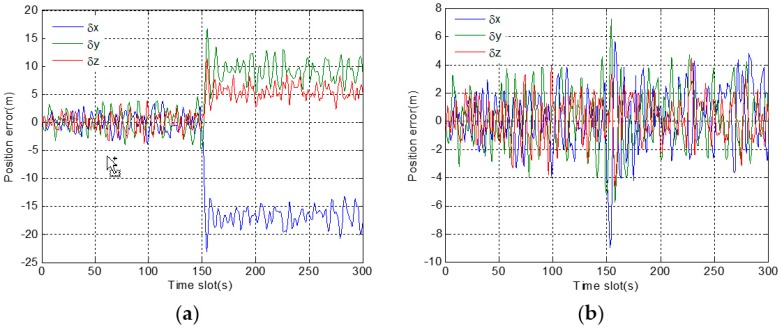
Position error in 3D direction corresponding to abrupt fault: (**a**) before using a robust algorithm; (**b**) after using a robust algorithm.

In Figure 10a, the position errors in a three-dimensional direction quickly deviate after fault injection. However, in Figure 10b, there is no great deviation except for a surge in the period of 151 s to 154 s. By comparing the two figures, it demonstrates that the proposed robust algorithm is also effective in dealing with the abrupt fault.

Even though the fault type changes, the robust algorithm based on CNN is still superior to that based on Huber by comparing the variances shown in Table 4. The proposed algorithm that doesn’t need any adjustment can output the desired factor adaptively according to the changes of fault, while the performance of Huber is restricted by its parameter. However, since the adjustment of the robust algorithm lags behind the rapid occurrence of abrupt fault, its variance is much larger than that without fault.

**Figure 11:**
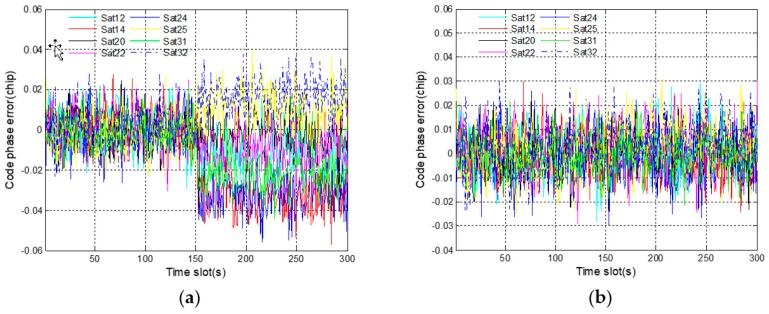
Code phase errors of eight satellite signals corresponding to abrupt fault: (**a**) before using a robust algorithm; (**b**) after using a robust algorithm.

As shown in Figure 11a, different deviations occur instantly in the code phase errors of all eight satellites after fault injection. The result proves once again that the fault propagation problem does exist in deep integration. Compared with the gradual fault, the abrupt fault is exposed faster and spreads faster to the other satellites. In Figure 11b, there is no deviation, proving that the fault propagation problem is solved by using the proposed robust algorithm.

**Figure 12:**
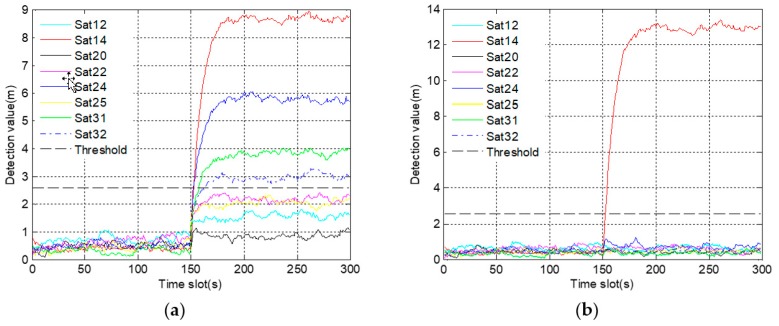
The graph of fault identification statistics corresponding to abrupt fault: (**a**) before using a robust algorithm, (**b**) after using a robust algorithm.

In Figure 12a, not only do the detection values of four satellites quickly exceed the alarm threshold, but also the time of exceeding the threshold is very close. These results make it difficult to identify the actual fault satellite, thus increasing the probability of misjudgment. On the contrary, it is easy to identify the satellite 14 as the fault satellite for the reason that only the detection values of satellite 14 exceed the alarm threshold in Figure 12b. Furthermore, 100 sets of simulation data are used for Monte Carlo test. The test result is that the accuracy of fault identification is up to 97%, which is increased by 25% after using a robust algorithm.

#### 4.2.2. Experimental Test

In cities, GNSS signal occlusion is a common phenomenon due to the blocked factors such as high buildings, tree shades, and tunnels. Occlusion can lead to signal loss, and then the output of the corresponding receiving channel is noise. If the fault channel cannot be identified and isolated from the system in time and accurately, the fault information will inevitably contaminate the navigation solution. The experimental system shown in Figure 6 is placed in the occluded situation to verify the robustness of deep integration. The test procedure is to place the trolley in an open area to complete the processes of searching for stars, positioning and INS alignment. After the system output is stable, the trolley is pushed along a high building. Then, it is pushed back to the starting point. The test lasts about 10 minutes for three round trips.

**Figure 13:**
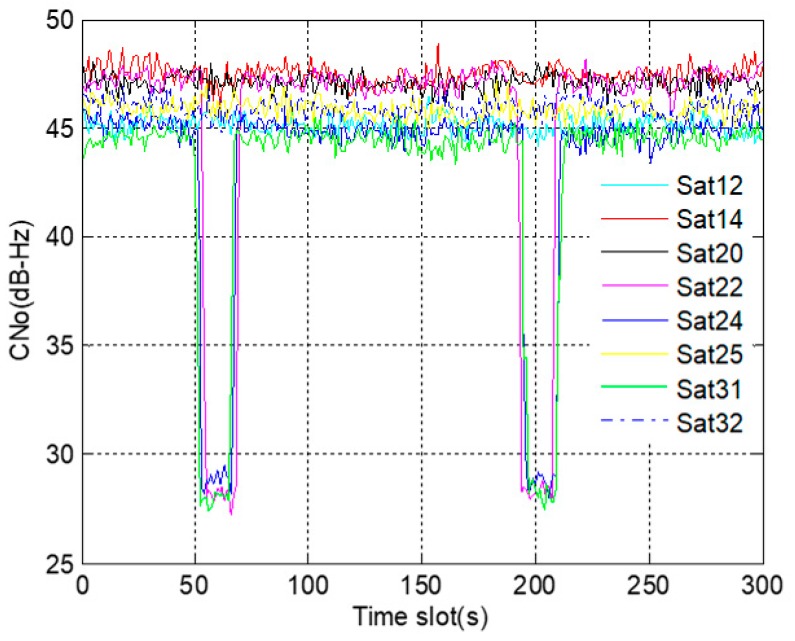
C/No of GNSS signals.

The data collected at one of the trips is utilized for analysis. Figure 13 shows the changing process of C/No of GNSS signals. As shown in the figure, the C/No of all GNSS signals was normal from the beginning to 50 s. Then, during the period of 51 s to 68 s, the signals of satellite 22, 24 and 31 were lost successively due to the occlusion of high building, and the corresponding C/No decreased rapidly. After passing the high building, the signals of the three satellites recovered one by one. Next, in the process of returning along the original route, the occlusion was experienced again. From 189 s to 208 s, the C/No showed a rapid decline, and the signals of satellite 22, satellite 24 and satellite 31 were lost for the second time.

**Figure 14:**
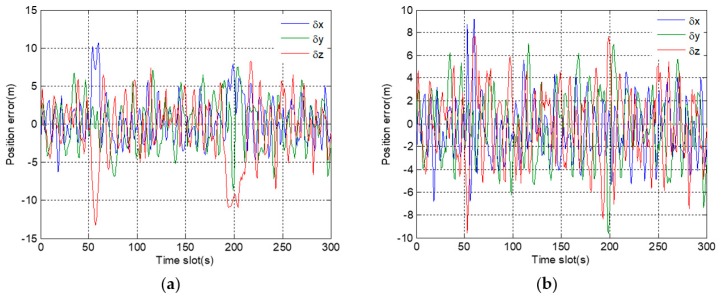
Position errors in 3D direction during occlusion: (**a**) before using a robust algorithm; (**b**) after using a robust algorithm.

In Figure 14a, the positioning results deviate greatly in the period of 51 s to 68 s and 189 s to 208 s. These two periods correspond to the time of two satellite signals loss. When three satellite signals are occluded, the system makes use of the receiving data of the remaining five satellites to maintain the output of positioning results and predict the code phase and carrier frequency of each satellite signal. Once the occluded GNSS signals recover, the system quickly captures these signals based on the predicted loop parameters, then the positioning results return to normal rapidly. After using the proposed robust algorithm, the positioning results are shown in Figure 14b. Although the position errors fluctuate slightly during the period of signal occlusion, the maximum deviation does not exceed 10 m, which is much smaller than the deviations in Figure 14a.

**Figure 15:**
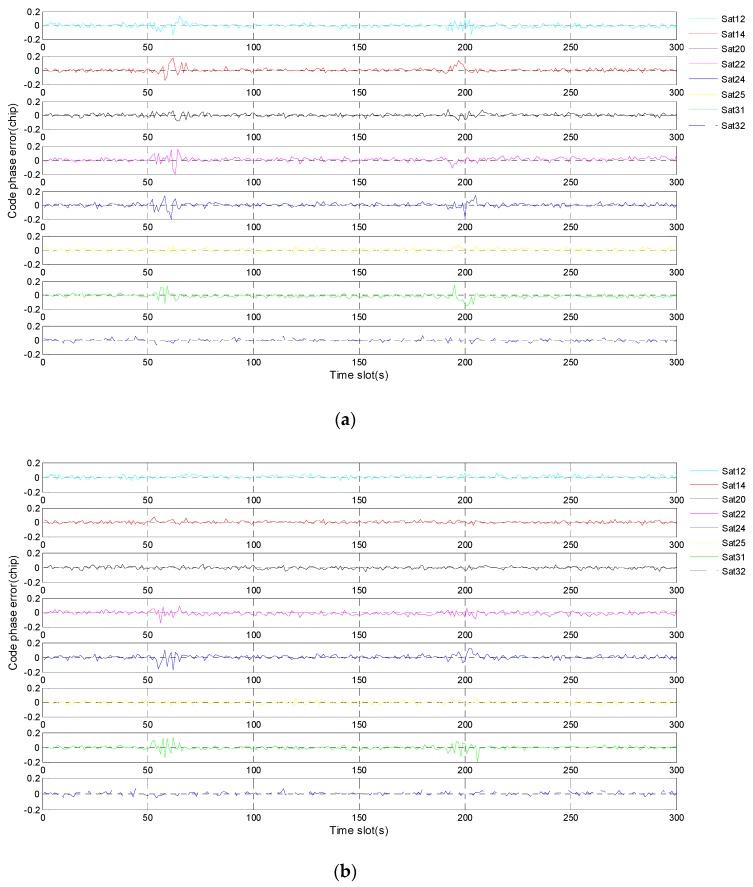
Code phase errors of eight satellite signals during occlusion: (**a**) before using a robust algorithm; (**b**) after using a robust algorithm.

Compared with Figure 15a, it can be found that only the three occluded satellites have deviations in Figure 15b, proving that the propagation of fault information is suppressed by the proposed robust algorithm. For the three occluded satellites, the deviations of code phase errors are due to the absence of normal input.

**Figure 16:**
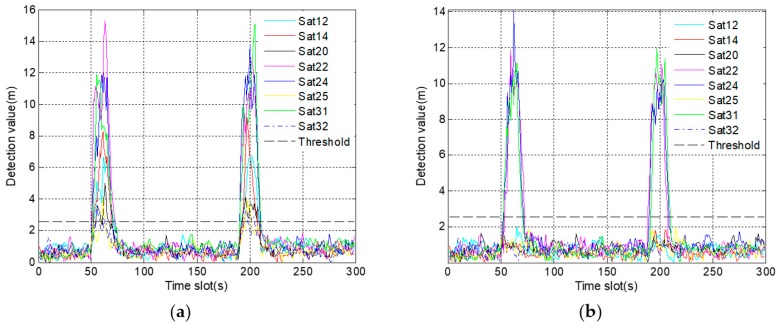
The graph of fault identification statistics during occlusion: (**a**) before using a robust algorithm; (**b**) after using a robust algorithm.

Because of the fault propagation problem, it is difficult to identify the occlude satellites from detection results in Figure 16a. However, in Figure 16b, only the detection values of the three occluded satellites exceed the alarm threshold during occlusion. Moreover, once the occluded signals return to normal, the corresponding detection values decrease rapidly, showing that the detection is accurate and timely.

**Figure 17:**
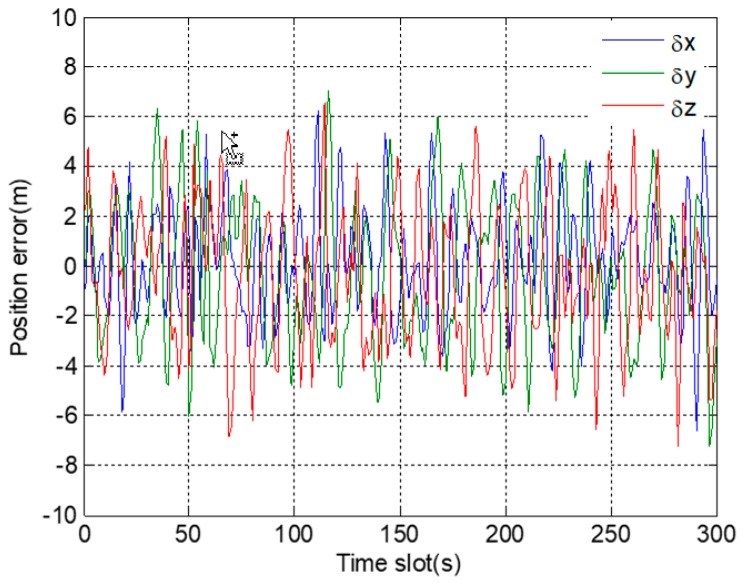
Position errors in 3D direction after isolating the fault channels.

In addition, it should be noted that the robust algorithm only has the ability to weaken the impact of fault information, but cannot completely eliminate the impact. If there are more fault channels, the adverse impact on navigation solution will be more serious. As shown in Figure 14b, even if the robust algorithm is utilized, a small surge still appears in the positioning results during signal occlusion. Considering the improvement that the fault channel can be identified with a high accuracy when using the proposed robust algorithm, it is preferable to remove the abnormal channel from the system according to the identification results. After the receiving channels corresponding to the occluded satellites are isolated from the system, the positioning results are shown in Figure 17. We can see that the position errors always remain within the normal range. The variance of position error δp is 2.25, while the variance of position error in Figure 14b is 3.95. The positioning accuracy is further improved after isolation.

## 5. Conclusions

In this paper, a novel robust algorithm which is based on CNN is proposed to be used in the deeply coupled GNSS/INS integrated system. It improves the accuracy of identifying the fault channel by solving the fault propagation problem. The validity of the proposed robust algorithm is verified by two types of experiments: artificial fault injection and outdoor occlusion. Whether it is the fault injection of a single satellite or the occlusion of multiple satellites, the fault propagation problem is confirmed to exist in the vector tracking loop. After using the proposed robust algorithm, the fault propagation problem is solved. As a result, there is no obvious deviation in the positioning results and code phase errors. The accuracy of gradual fault identification is increased by 21%, and the accuracy of abrupt fault identification is increased by 25%. The experiment results prove that the fault identification ability of deeply integrated GNSS/INS system is improved.

## Figures and Tables

**Table 1:** The configuration information of convolutional neural network (CNN).

Layer Name	Kernel/Subsampling Size	Output Size
Input layer	NA	2 × 10
Convolutional layer C1	3@2 × 5	3@2 × 6
Pooling layer P1	1 × 2	3@2 × 3
Convolutional layer C2	6@2 × 2	6@2 × 2
Pooling layer P2	1 × 2	6@2 × 1
Full-connected layer	NA	12 × 1
Output layer	NA	40 × 1

**Table 2:** Parameters of Micro-Electro-Mechanical System (MEMS).

Parameter Name	Accelerometer	Gyro
bias error (in-run)	±40 μgal	100 °/h
bias error (initial)	±2 mgal	±0.25 °/s
scale factor stability	±0.05%	±0.05%
output frequency	100 Hz	100 Hz
sampling rate	30 kHz	30 kHz

**Table 3:** Variance of position error corresponding to gradual fault.

Method	Variance (m)
No fault	1.802
CNN	2.070
Huber (d = 1)	2.517
Huber (d = 1.25)	2.358
Huber (d = 1.5)	2.832

**Table 4:** Variance of position error corresponding to abrupt fault.

Method	Variance (m)
No fault	1.802
CNN	3.157
Huber (d = 1)	3.606
Huber (d = 1.35)	3.411
Huber (d = 1.5)	3.725
